# LIBS-Acoustic Mid-Level Fusion Scheme for Mineral Differentiation under Terrestrial and Martian Atmospheric Conditions

**DOI:** 10.1021/acs.analchem.1c04792

**Published:** 2022-01-12

**Authors:** César Alvarez-Llamas, Pablo Purohit, Javier Moros, Javier Laserna

**Affiliations:** UMALASERLAB, Departamento de Química Analítica, Universidad de Málaga, C/Jiménez Fraud 4, Málaga 29010, Spain

## Abstract

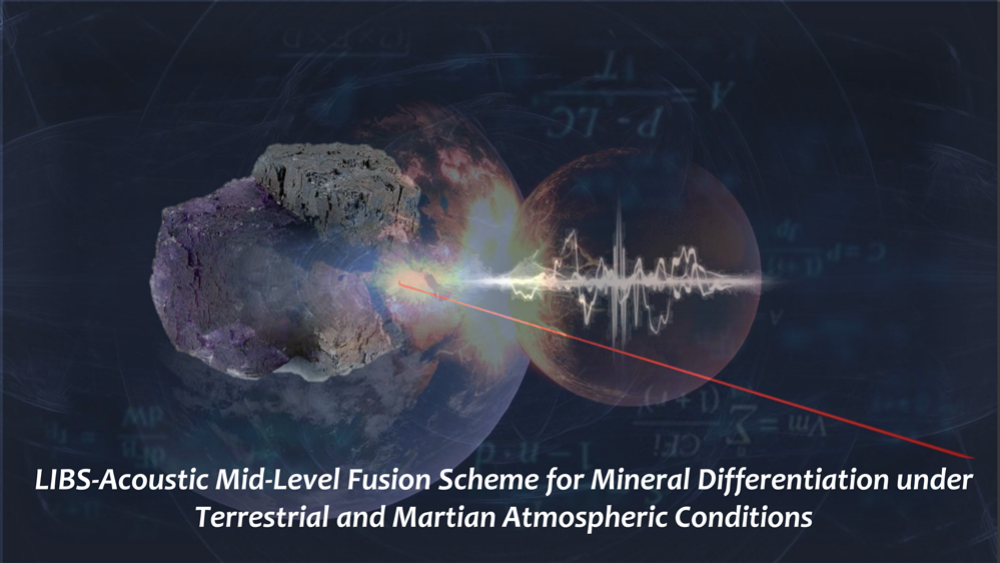

The shockwave produced alongside the plasma during a laser-induced breakdown spectroscopy event can be recorded as an acoustic pressure wave to obtain information related to the physical traits of the inspected sample. In the present work, a mid-level fusion approach is developed using simultaneously recorded laser-induced breakdown spectroscopy (LIBS) and acoustic data to enhance the discrimination capabilities of different iron-based and calcium-based mineral phases, which exhibit nearly identical spectral features. To do so, the mid-level data fusion approach is applied concatenating the principal components analysis (PCA)-LIBS score values with the acoustic wave peak-to-peak amplitude and with the intraposition signal change, represented as the slope of the acoustic signal amplitude with respect to the laser shot. The discrimination hit rate of the mineral phases is obtained using linear discriminant analysis. Owing to the increasing interest for in situ applications of LIBS + acoustics information, samples are inspected in a remote experimental configuration and under two different atmospheric traits, Earth and Mars-like conditions, to validate the approach. Particularities conditioning the response of both strategies under each atmosphere are discussed to provide insight to better exploit the complex phenomena resulting in the collected signals. Results reported herein demonstrate for the first time that the characteristic sample input in the laser-produced acoustic wave can be used for the creation of a statistical descriptor to synergistically improve the capabilities of LIBS of differentiation of rocks.

Laser–matter interaction encompasses a large number of phenomena taking place over a time lapse from femtoseconds to milliseconds.^1,2^ The rapid succession in which events unfold after the laser light reaches the sample turns this process into a complex scientific problem. Still, as demonstrated by decades of research, thoroughly comprehending the different stages from the absorption of laser photons and changes in the optical traits of surface to thermal relaxation, material ablation, and plasma formation is of great interest for analytical applications. Laser-induced breakdown spectroscopy (LIBS) takes advantage of the elemental and molecular emission to reveal physicochemical information pertaining to the inspected sample.^3,4^ Currently, LIBS is a mature technique that can face the chemical characterization of a variety of samples inaccessible to a majority of other analytical tools.^5^ To name a few figures of merits, the scarce amount of sample required allowed the analysis of single nanoparticles with masses down to the attogram scale.^6^ The capability to carry out simultaneous multielemental characterization with a high spatial resolution has been exploited for a wide range of targets including biological, industrial, and geological samples.^7^ Additionally, fieldable and remote sensors are a key application niche for LIBS where it truly exhibits its adaptability,^8^ from submerged shipwrecks,^9^ through geochemical in-field analysis,^10^ to space exploration.^11^

Shockwave generation is one of the phenomena occurring alongside the laser-induced plasma owing to the explosive ejection of ablated material into the surroundings. The plasma shockwave is expected to encompass two different, entwined contributions: one related to the expansion of the plasma, and another linked to the mechanical relaxation of the probed material. Despite the interest shown previously in exploring laser-induced acoustics as a means to expand the information delivered by LIBS inspection of solids, the multiple sources of uncertainty linked to sound waves have hindered their potential application in sensors for both in-lab and off-lab studies. Nonetheless, the recent integration of a microphone synchronized to the LIBS laser in the SuperCam instrument in the Perseverance rover deployed as part of the NASA Mars 2020 mission has re-kindled the motivation toward unraveling the shockwave as it evolves in time to extract the samples’ contribution from it seeking to ascertain parameters such as the crystalline structure and the presence of layers or to enhance the overall SuperCam performance.^12−14^

Early reports on laser-induced acoustics outlined the relation existing between acoustic energy and the energy density at the sample^15^ and the possibility to discriminate the mechanism leading to plasma formation on the basis of the recordings with further research being focused on using acoustics to normalize LIBS data.^16^ More recently, a series of studies by Murdoch et al. and Chide et al. set the basis for modern-day laser-induced acoustics while performing tests for the SuperCam instrument.^14,17^ These authors carried out the recording of plasmas formed under Earth and Martian atmospheric conditions (i.e., about 7 mbar CO_2_, *T* down to −80° C at the microphone and different intensity wind currents) on Martian simulant targets as well as in minerals of interest for the mission. Results indicated a strong dependence of the generated acoustic wave with physical properties such as the hardness of the material, and therefore the ablation rate, the density, and the thermal conductivity. Sound propagation as a function of the medium and the length of the acoustic path was also evaluated in refs (17, 18) since the sound wave is known to strongly attenuate with distance and the LIBS + acoustic tandem is intended to be used to measure samples located 2–7 m away from the rover. In spite of positive results indicating the complementarity of acoustics and additional information it can provide, many questions still remain as to how LIBS data, specifically for the identification and classification of mineral phases, can be improved by merging of simultaneously acquired sets.

In the present work, Fe-based and Ca-based minerals featuring intraseries similar LIBS spectra are probed at a sampling distance of 2 m under Earth and Mars-like atmospheres. LIBS data and simultaneously acquired acoustic recordings were pre-processed to develop a mid-level data fusion strategy, based on a combination of LIBS principal components analysis (PCA) and acoustic features to create a new sample descriptor allowing better differentiation of samples with extremely similar LIBS spectra. After the model was verified for mineral data acquired in Earth atmosphere, the training set was sampled under Mars-like conditions. Results indicate that, under a tightly controlled experimental scenario, acoustic data in the time domain can be merged with LIBS spectra to enhance the discrimination capabilities of the technique owing to differences found in the laser-produced sound wave, thus paving the way toward extracting relevant chemical data from this newly exploited source of information.

## Experimental Section

### Experimental Setup

**Figure 1:**
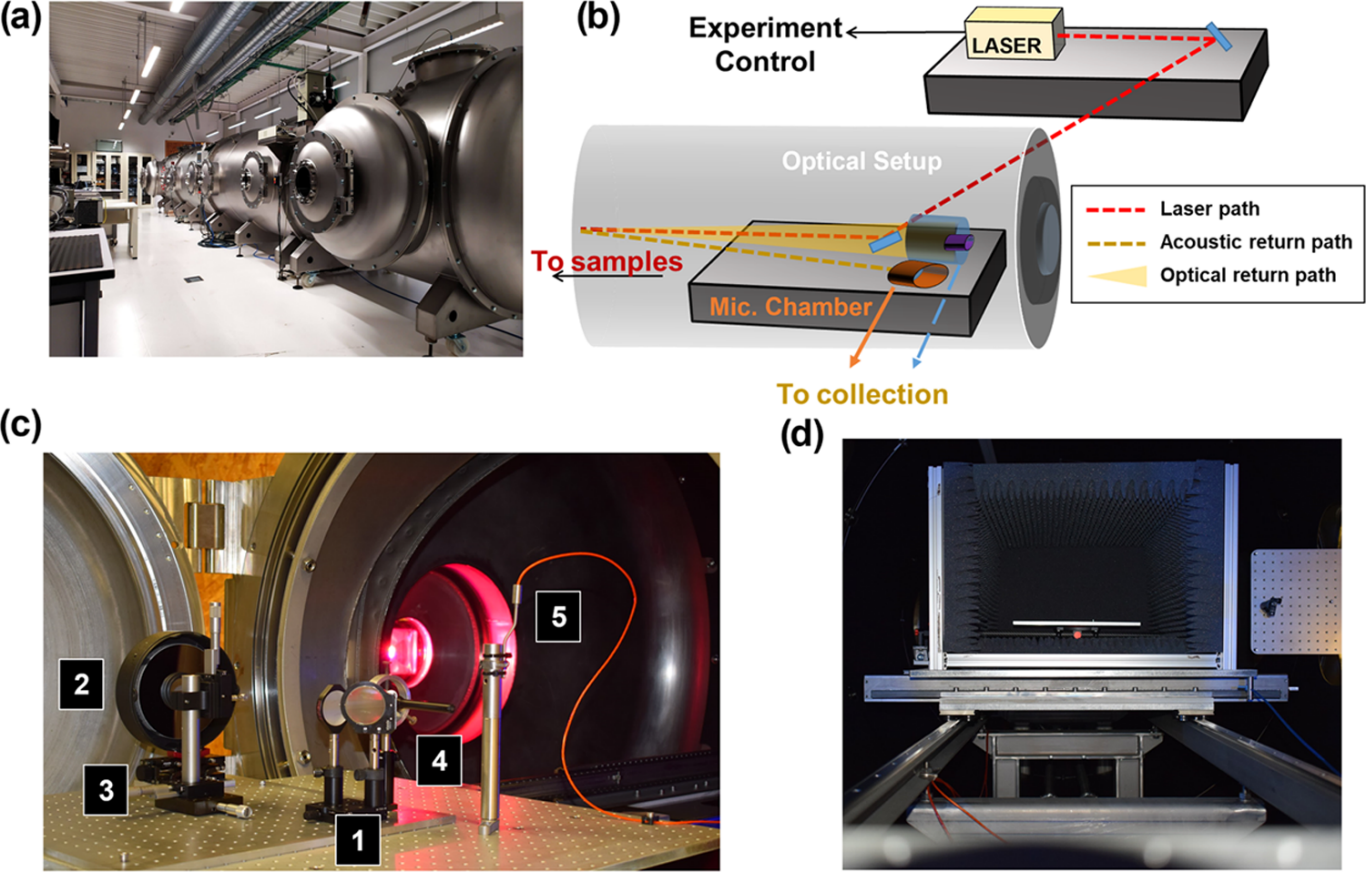
(a) Panoramic view of the TVC in the UMALASERLAB. (b) Experimental setup scheme. (c) Optical setup placed inside the TVC: 1, Laser mirror and telescope’s secondary mirror; 2, Telescope’s primary mirror; 3, Optical fiber holder; 4, Microphone without its hemi-anechoic chamber; 5, Temperature monitoring thermocouple. (d) Sample holder inside the hemi-anechoic chamber.

Experiments reported herein were performed in both terrestrial and Martian atmospheres. Measurements were conducted inside a thermal vacuum chamber (TVC) installed at the UMALASERLAB (Figure 1a). This facility was built by AVS—Added Value Solutions (Gipuzkoa, Spain) for remote spectroscopic studies in Earth and extraterrestrial environments. The TVC is a 12 m long and 2 m diameter cylinder with a total useful volume of ca. 24 m^3^ capable of recreating the atmospheric conditions of different planetary bodies within a range of 200–400 K and 10^–3^–10^3^ mbar.^19^ The chemical composition of the working atmosphere could also be regulated by a gas inlet using a mass flow controller.

**Figure 2:**
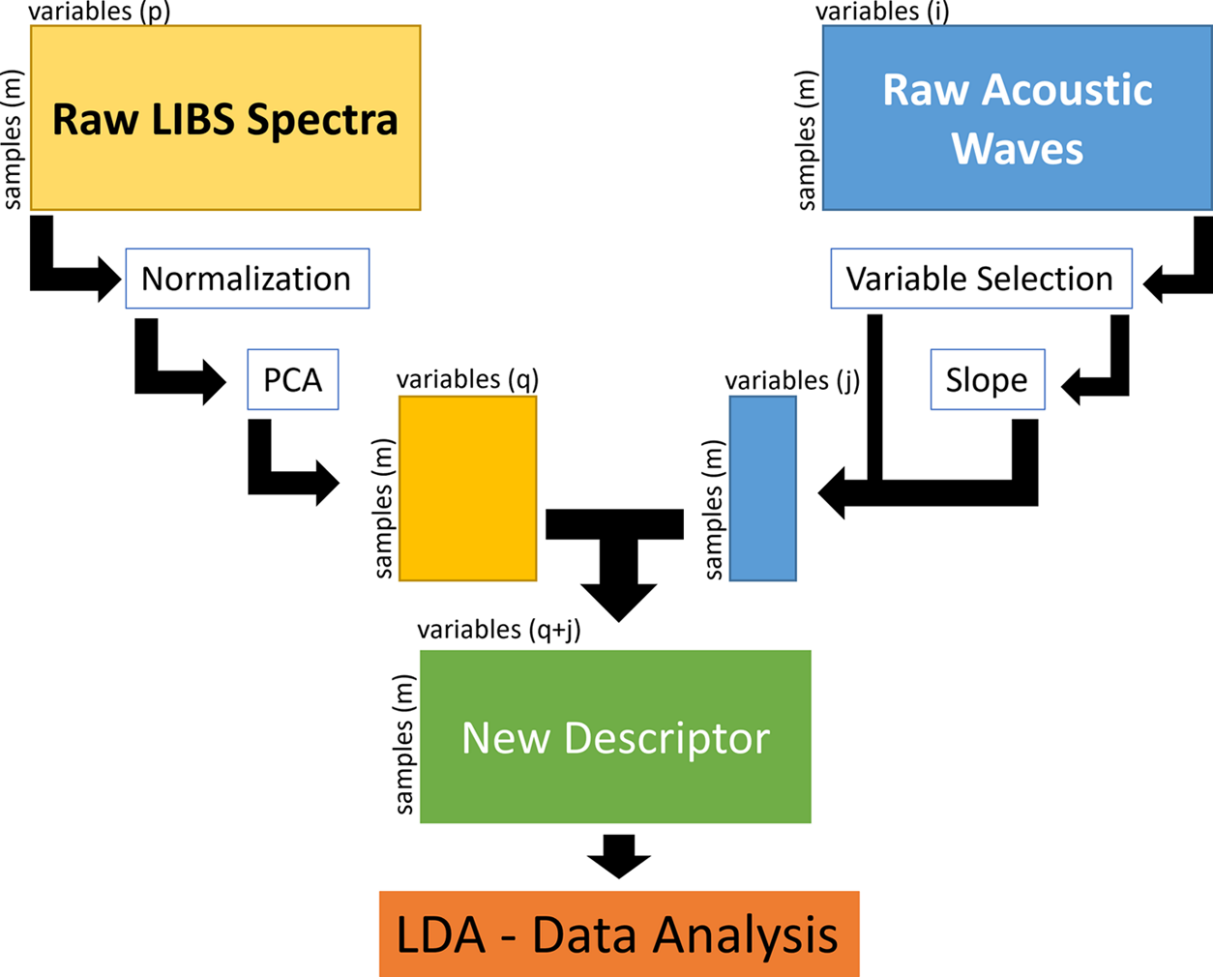
Data fusion process scheme.

**Figure 3:**
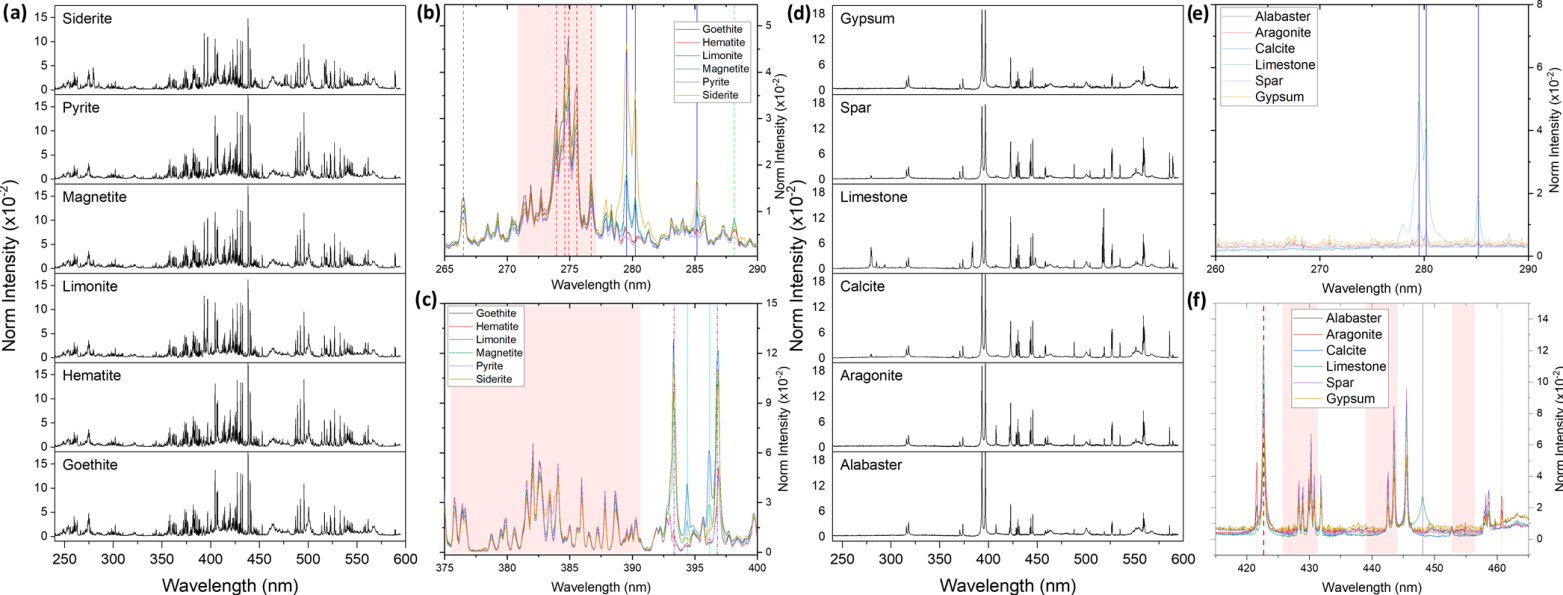
Average LIBS spectra for the analyzed samples remarking the presence of some detected impurities. (a) Average spectra of Fe-rich minerals, (b) spectra (265–290 nm) showing emission lines of Fe II-red dashed/shadow, Mg I and II-blue, and Si I-green dashed. (c) Spectra (375–400 nm) with Fe I-red shadow, Ca II-purple dashed, and Al II-bright blue. (d) Average spectra of Ca-rich minerals. (e) Spectra (260–290 nm) with Mg I and II. (f) Spectra (415–465 nm) with Ca I-red dashed/shadow and Sr I and II-orange dashed, and Mg II.

A schematic diagram of the experimental setup is presented in Figure 1b. A pulsed Nd:YAG laser (Ekspla, model NL 303D/SH; @1064 nm, 45 mJ, pulse width 4 ns) located outside the TVC was used as the excitation source. Collimated laser pulses were expanded and then focused using a beam expander prior to entering the TVC through a borosilicate glass vacuum viewport. Once inside, laser pulses were guided toward the target (Figure 1c-1) for generating the recorded plasmas. Samples were placed upon a platform coupled to a motorized linear stage allowing displacement in the axis perpendicular to the laser path (Figure 1d). Plasma emission was remotely collected using a 6′ Ritchey–Chrétien telescope (f/9, focal distance 152 mm) modified to integrate an optical fiber (Figure 1c-1–3). The plasma image was coupled to a 1000 μm single optical fiber, which was bifurcated after the fiber optic vacuum feedthrough to SMA terminated 600 μm fibers, with a total length of 3 m. A pair of miniature Avantes Czerny-Turner spectrographs with a 75 mm focal length placed outside the TVC was also used with a fixed integration time of 1.28 μs. Under this configuration, LIBS spectra ranging from 240 to 590 nm were recorded.

**Figure 4:**
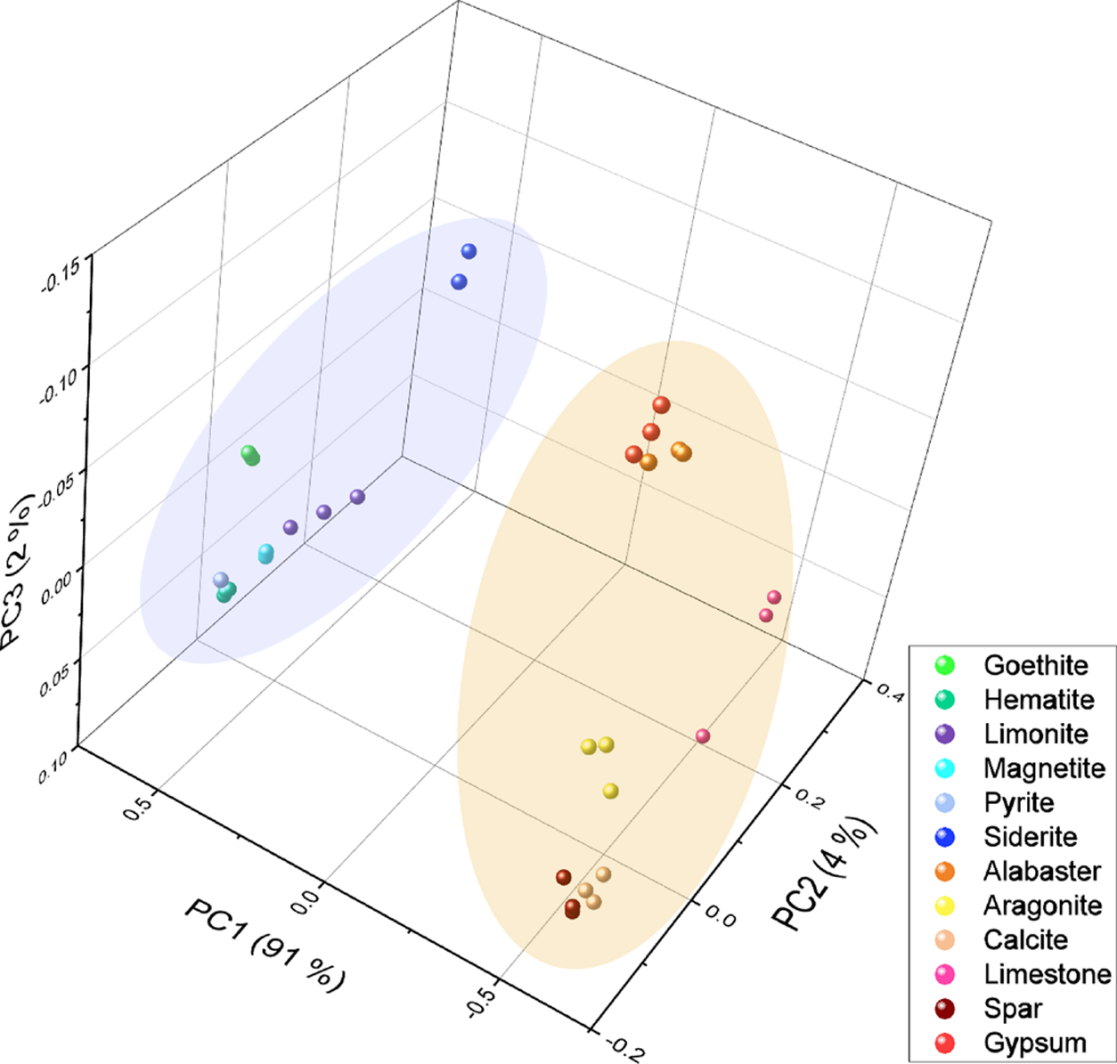
PCA scores for the first three PCs. Cold tones correspond to Fe-rich materials, while warm tones correspond to the Ca-rich subgroup.

Acoustic waves coming from laser ablation and plasma expansion were recorded using a 6 mm pre-polarized condenser microphone (20 Hz to 19 kHz frequency response, omnidirectional polar pattern, 14 mV·Pa^–1^ sensitivity, TR-40 model from Audix). The microphone (Figure 1c-4) was housed inside a custom-built hemi-anechoic chamber (50 cm length, 25 cm internal diameter) with inner and external walls insulated using acoustic foam. This acoustic suite was positioned at a fixed sample surface-to-microphone distance of 2 m. The source–receiver path featured an angle of about 7° to avoid obstructions in the optical path. A 24-bit/192 kHz audio interface (UA-55 Quad-capture, Roland) was used at a sampling rate of 96 kHz for the digitalization of acoustic waves. Audacity software was employed as an audio recording application. The sample holder was placed inside another custom-built hemi-anechoic chamber (70 × 40 × 40 cm^3^, L × W × H—Figure 1d) covered with HiLo-N40 acoustic foam made of polyurethane with high rigidity and low density (70 mm total thickness, 40 mm knob height, 16.5 kg·m^–3^ bulk density). This optical configuration led to a spot diameter of ca. 300 μm at the surface of samples located at 2 m distance away from the last mirror and the collection optics. To avoid acoustic reflections, the ground between the samples and the microphone was also covered with anechoic foam.

### Samples

To evaluate the input of the acoustic response to the information yielded by laser-induced plasmas and better identify geological specimens, 12 minerals, 6 rich in Fe and 6 rich in Ca, were selected. These minerals were chosen as their expected elemental composition should translate into very similar LIBS spectra, either due to the presence of some components in the atmosphere, thus making its source ambiguous, or due to their difficult detectability by LIBS (e.g., S).

Pyrite (FeS_2_), siderite (FeCO_3_), magnetite (Fe_3_O_4_), hematite (Fe_2_O_3_), limonite (FeO(OH)*_n_* H_2_O), and goethite (FeO(OH)) were selected as Fe-bearing minerals. Iron is among the 18 most abundant elements in our solar system, and, particularly considering the interest in Mars exploration, these specimens have been both detected in Martian meteorites and identified in situ in several regions on the Red Planet.^20−22^

Moreover, a group of six calcium-rich minerals was evaluated. Calcium is a key constituent of rock-forming minerals and can yield clues about the material that formed the Solar System’s rocky planets (Mercury, Venus, Earth, and Mars). In this context, limestone, a sedimentary rock consisting primordially of calcium carbonate, alongside aragonite, calcite spar, and calcite, all with the same expected composition (CaCO_3_), were probed owing to their close LIBS response. Finally, gypsum, common sulfate mineral composed of hydrated calcium sulfate (CaSO_4_·2H_2_O) and its fine-grained massive variety, called alabaster (CaSO_4_·2H_2_O), were also investigated.^22−26^

Prior to their analysis, the minerals, originally in their natural form, were cut and polished. A total of 75 laser events were analyzed, i.e., 25 plasma events per position at three different positions along the surface of the mineral.

### Data Processing

A new global descriptor was created to perform data fusion via a combination of the spectral responses from both the laser-induced plasma emission and the acoustic wave, to describe the interrogated targets. This combination can be done at different levels: low-level—working with the spectra by simply concatenating or applying the outer vector over the responses; mid-level or intermediate fusion—for which some relevant features from each source are treated independently and then concatenated into a single array used for multivariate classification; and high-level fusion—the classification or regression models are run from each data array, and the results are combined to obtain the final result.^27^ The second approach was applied in this work.

It should be stressed that, although LIBS and acoustic responses are originated from the same laser event, the mechanisms governing each wave differ. In LIBS, the signal mainly derives from atoms (neutrals or ions) that have sequentially undergone a process of ablation, fragmentation, atomization, ionization, and excitation, in a proper temporal range, and at a wavelength range detectable by the spectrometer. In the case of acoustics, the main wave is attributed to the compression/rarefaction generated due to plasma expansion. For this reason, the combination of both data can provide complementary information to obtain this new attribute aimed to identify the interrogated specimen more clearly. Figure 2 shows the full data fusion scheme applied. In the intermediate data fusion scheme followed herein, different features were extracted independently from each emission and sound wave spectra data set. In the case of LIBS spectra, data were averaged to convert a set of 12 × 3 × 25 (samples × positions × spectra number) into a 12 × 3 one, generating an average spectrum for each position. Therefore, an ***n*****×*****j*** matrix was obtained, where ***n*** corresponds to the number of median spectra and ***j*** corresponds to the number of spectral variables. These median spectra were normalized using unit vector normalization (eq 1) and mean centering (eq 2).
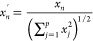
1
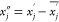
2

Principal component analyses were performed using the resulting matrices. PCA provided the weights needed to get the new variable (principal component)^28,29^ that best explained certain underlying facts from a multivariate data set without diluting essential information. Therefore, the PCA served not only as a variable reduction step but also as exploratory data analysis (EDA), being a zero step prior to the supervised classification model.^30^ In the acoustic dataset, two different features were extracted from the time domain responses: the wave peak-to-peak amplitude and the variation of this value for a specific position. Both values are linked to the physical traits of the material,^14,17^ supplying complementary information to LIBS spectra. Then, the PCA score matrix and the acoustic features were concatenated, obtaining the new descriptor in the mid-level fusion process. This descriptor was used as an input matrix for studying the classification’s throughput for the minerals using linear discriminant analysis (LDA). LDA creates a linear boundary between classes based on the distance to each class’ centroid. This is calculated using the Mahalanobis distance.^30^ All of the data analysis described in this subsection was performed using Matlab 2020b, Mathworks, Inc. software.

## Results and Discussion

### Exploratory Data Analysis on LIBS Spectra

First, exploratory data analysis on the emission spectra obtained by LIBS was performed. Figure 3 shows the average spectra for all of the samples (Figure 3a,d). As expected, LIBS information of minerals containing line-rich elements (e.g., Fe or Ca) exhibit almost identical spectral features. The minimal differences detected between collected LIBS data may be attributed to fluctuations in the morphological and physical characteristics of the material guiding the ablation rate and altering the laser-matter coupling rather than to changes in the concentration of the constituent elements. Moreover, as observed in Figure 3, the emission of some minor or unexpected elements was present in the spectra (Figure 3b,c,e,f). For Fe-rich minerals, these lines were mainly attributed to Mg, Ca, and Si, whereas for Ca-rich minerals, Mg and Sr were the most relevant impurities. As discussed in the Experimental Section, PCA was applied on the median LIBS spectrum for each position after continuum background subtraction, normalization, and mean centering. Figure 4 presents the score values for the first three principal components, with a total explained variance of 97%. From Figure 4, it is readily apparent how the two subgroups studied were clearly separated through the PCA score three-dimensional (3D) graph. This was expected due to the notorious compositional differences found between the two subgroups. A partial, yet incomplete, separation of each mineral phase within each of the subgroups can be observed. This behavior is justified by the presence of impurities in each mineral, as shown in Figure 3, that create the cluster pattern observed in Figure 4.

### Exploratory Data Analysis on Acoustic Data

**Figure 5:**
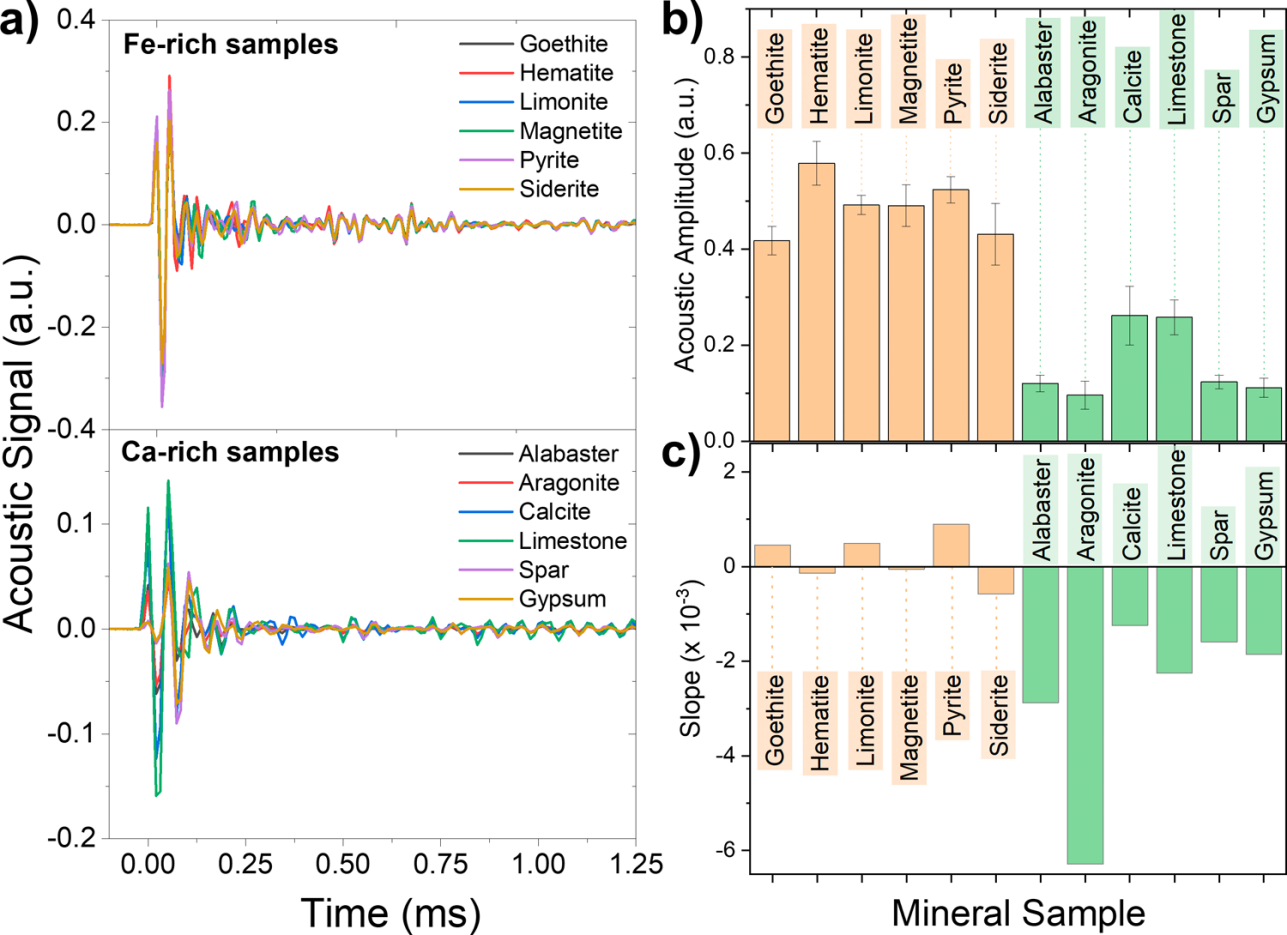
(a) Average time domain signals in the 0.1–1.25 ms range. (b) Average amplitude in the first maximum of the acoustic wave for each mineral studied. (c) Slope value obtained for each mineral. Values closer to 0 indicated stable signal during the laser shot series, while negative values implied decreases in the measured sound intensity.

The acoustic waves produced by the laser-induced plasma detonation were recorded simultaneously with the LIBS spectra. Figure 5a represents average spectra in the time domain. The spectra were aligned to the first maximum observed, arbitrarily defining this point as *t* = 0 ms. The evolution of the acoustic waves with time was found to have a matching behavior for all samples, with a duration from the first maximum below 0.5 ms. Features at longer times were associated with the formation of echoes in the TVC. Echoes are bound to be formed due to sound wave collisions with the inner walls of the chamber or due to reflections with the elements composing the setup such as optical and optomechanical parts. Since an open-air path connected the two hemi-anechoic chambers, some of these reflections could be recorded by the microphone. Still, the different pathlengths traversed by reflected sound waves, echoes, and the original plasma blast were long enough to separate these contributions in time, thus allowing us to clearly temporally differentiate them and to choose only the portion directly related to the sample. Therefore, the information provided by the first maximum (*t* = 0 in Figure 5) of the acoustic wave should be completely free from interferences.

As mentioned, two different values of interest were extracted from the peak-to-peak amplitude, its total value, and the coefficient of intensity variation as the material was drilled by the laser in one sampling position. This coefficient, given by the slope of the amplitude as a function of the number of shots, provides the change of the acoustic signal when drilling down into the sample due to successive laser shots. Figure 5b,c shows the variation of the average amplitude in the first maximum of the acoustic wave for each mineral studied (Figure 5b) and of the slope (Figure 5c), showing a clear difference between the two subgroups. The values obtained in Figure 5 point toward a clear difference between the Ca and Fe groups, with similar values inside each group. Furthermore, the negative slope present for the Ca group indicated lower hardness and higher ablation rate per sample.^14,17^

### Data Fusion Treatment

**Figure 6:**
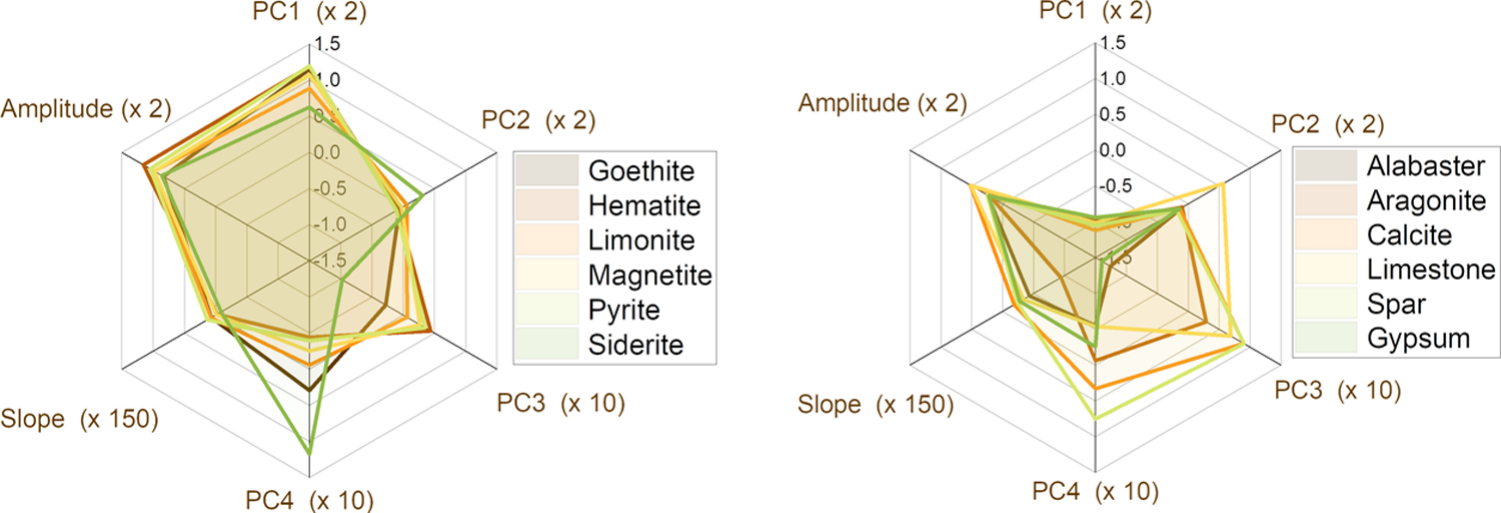
Graphical representation of the new LIBS-acoustic descriptor for Fe-rich minerals (left) and Ca-rich minerals (right). A factor is applied to each element to improve the visualization.

The mid-level fusion step was carried out taking into consideration the descriptors obtained during the exploratory analysis described in the previous sections. From the LIBS point of view, four PCs were selected (>97% of cumulative percentage of the total explained variance). Therefore, the new fused descriptor was constructed by concatenating the score arrays from the LIBS-PCA with the values coming from the amplitude of the acoustic wave and the slope of its decay at each sampling position. This new descriptor is graphically represented in Figure 6, allowing us to clearly differentiate between the two main subgroups. Each group presented a characteristic graphical trait, with the Fe-rich minerals being the more intense in the acoustic amplitude and in the PC1 score. This PC showed more relevant presence of Fe lines in the PCA loadings, while the Ca-related scores had negative scores. PC2 and PC4 seemed to have similar values for all of the samples, exhibiting less subgroup-dependent behavior and, probably, reporting information for the intragroup sample clustering. The acoustic input here is equivalent to that seen in Figure 5a,b.

**Figure 7:**
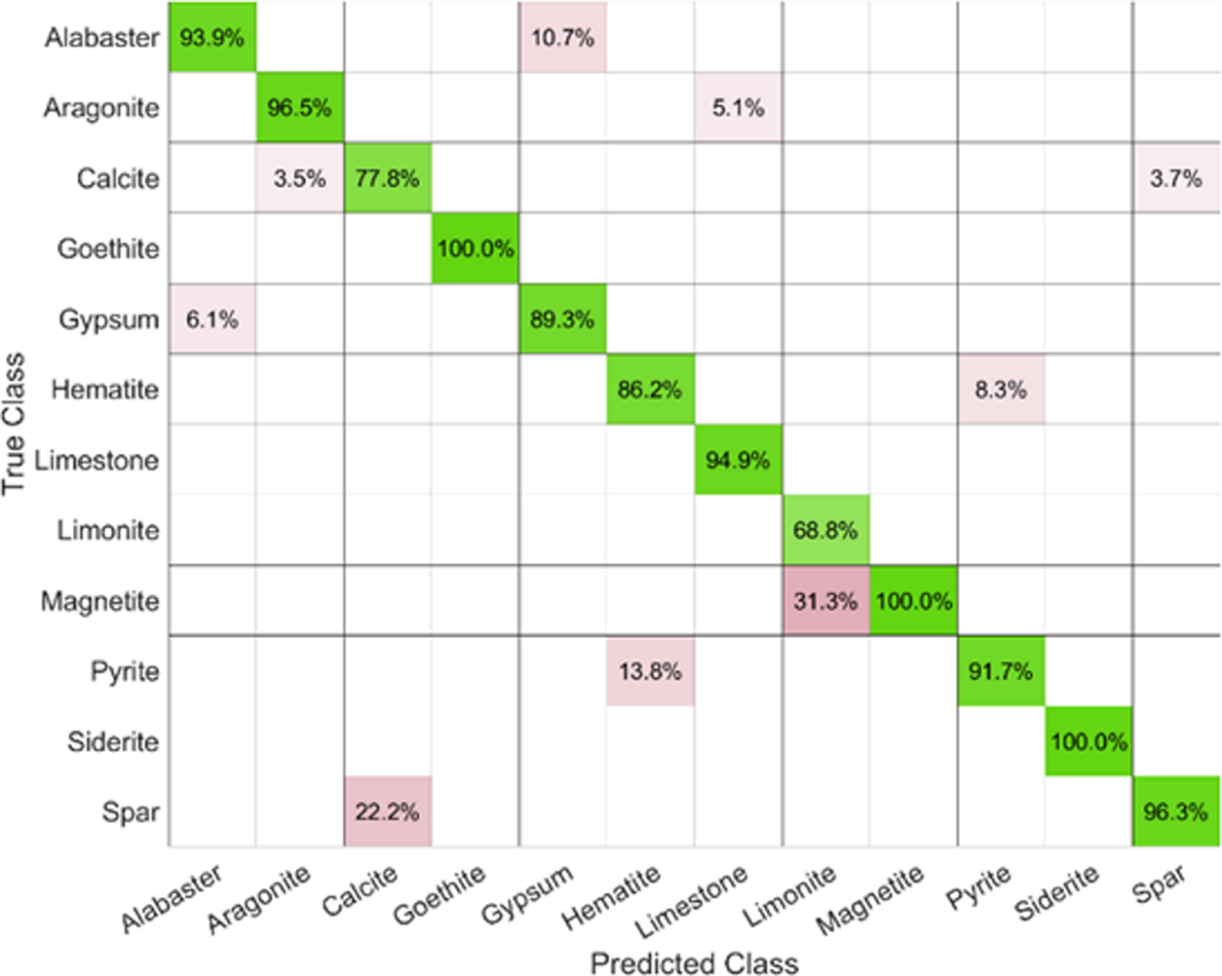
Percentage confusion matrix for the studied samples.

Once the new descriptor was built, it was evaluated to assess its feasibility to discriminate different minerals according to the fused optical and acoustic features. The classification performance of this new descriptor was, therefore, analyzed using an LDA model on the samples descriptors. The hit rate was individually calculated as follows for each data set: The data matrix, composed of the new descriptors built for the different sampling positions in each sample, was randomly divided into two matrices, i.e., the training matrix (75% of the samples) and the validation matrix (25%). Since there were only three sampling positions per sample, we imposed that there were no more than one replicate per sample in the validation matrix. By doing so, samples were not underrepresented in the training matrix. Subsequently, the LDA model was obtained using only the training matrix, and this model was then applied to the validation matrix to obtain the predicted group. These predictions were compared to the actual class values for the training matrix and the validation matrix. This process was iteratively repeated 1000 times with the resulting prediction values being the average of the percentage of correct assignations for all iterations. Due to the randomness of the matrix selection process, a limitation to prevent two different iterations from having the same components was also imposed. Table 1 shows the success rate for each data source, for the fused data and the original ones. LIBS success rate was obtained using four PCs (LIBS-PCA column) and the original LIBS spectra (normalized and mean-centered). Training and validation data are also represented, providing the training matrix better predictions, as expected. It is clear that fused data improved the classification capabilities upon comparison with the original data. Acoustic features on their own provided the worst hit rate between the studied cases. Still, the hit rate was above 74% in all of the studied cases. Also, LIBS spectral-related hit rate slightly overcame the LIBS-PCA, due to the presence of more qualitative information in the original spectra than in the PCA’s score values. Furthermore, the intragroup success rate was also calculated, that is, using only samples belonging to one of the two inspected families being considered. Figure 7 shows the confusion matrix for the obtained results from the fused data. Figure S1 in the Supporting Information presents the independent confusion matrix for LIBS-PCA and acoustics data. For the Ca-rich samples, the more common false prediction was related to extremely similar mineral phases (i.e., alabaster-gypsum or aragonite-spar-calcite), while the main source of error in Fe-rich samples corresponded to the magnetite-limonite pair. Both minerals had similar average contributions from Mg, Si, and Ca elemental emission lines, so failures in the assignation may be attributable to these spectral presences. No false identification between the different subgroups was observed due to the dramatic differences in both emission spectral and acoustical responses reported.

**Table 1:** LDA Hit Rate Obtained in Earth Atmosphere of the New Descriptor (Fusion), LIBS, and Acoustic Data

	LIBS (%)	LIBS-PCA (%)	acoustic (%)	fusion (%)
training	99	98	96	99
validation	91	90	77	92
Fe-based	89	95	82	92
Ca-based	93	89	74	87

### Application of the Data Fusion Strategy to Mineral Information Collected under a Martian Atmosphere

The preceding sections have shown that the mid-level fusion of optical emission and acoustic data from the laser-induced plasmas improves the classification capabilities for mineral phases in comparison to LIBS data alone. As a further step in this work, the developed strategy was applied to the same sample set under Mars-like atmosphere. Mars atmospheric pressure varies between 6 and 10 mbar depending on the geographical area and the season.^31^ The composition of the Mars atmosphere was determined by the Viking landers in the 1970s. Carbon dioxide is the main constituent (95%), followed by nitrogen (2.7%), argon (1.6%), and others (<0.5%).^32^ The parameters in the current experimental atmosphere were defined as 8 mbar of a CO_2_-rich atmosphere (>95%) with a sample temperature of 273 K.

The changes in the behavior of the laser-induced plasma caused by variable atmospheric pressure and composition have been widely reported;^33−35^ however, the propagation of plasma-induced pressure waves is less known. Changes in the transmission of the acoustic wave (which will depend on *P*, *T*, and composition of the wave’s path) were expected both in sound intensity and in the specific absorption of a range of frequencies.

**Figure 8:**
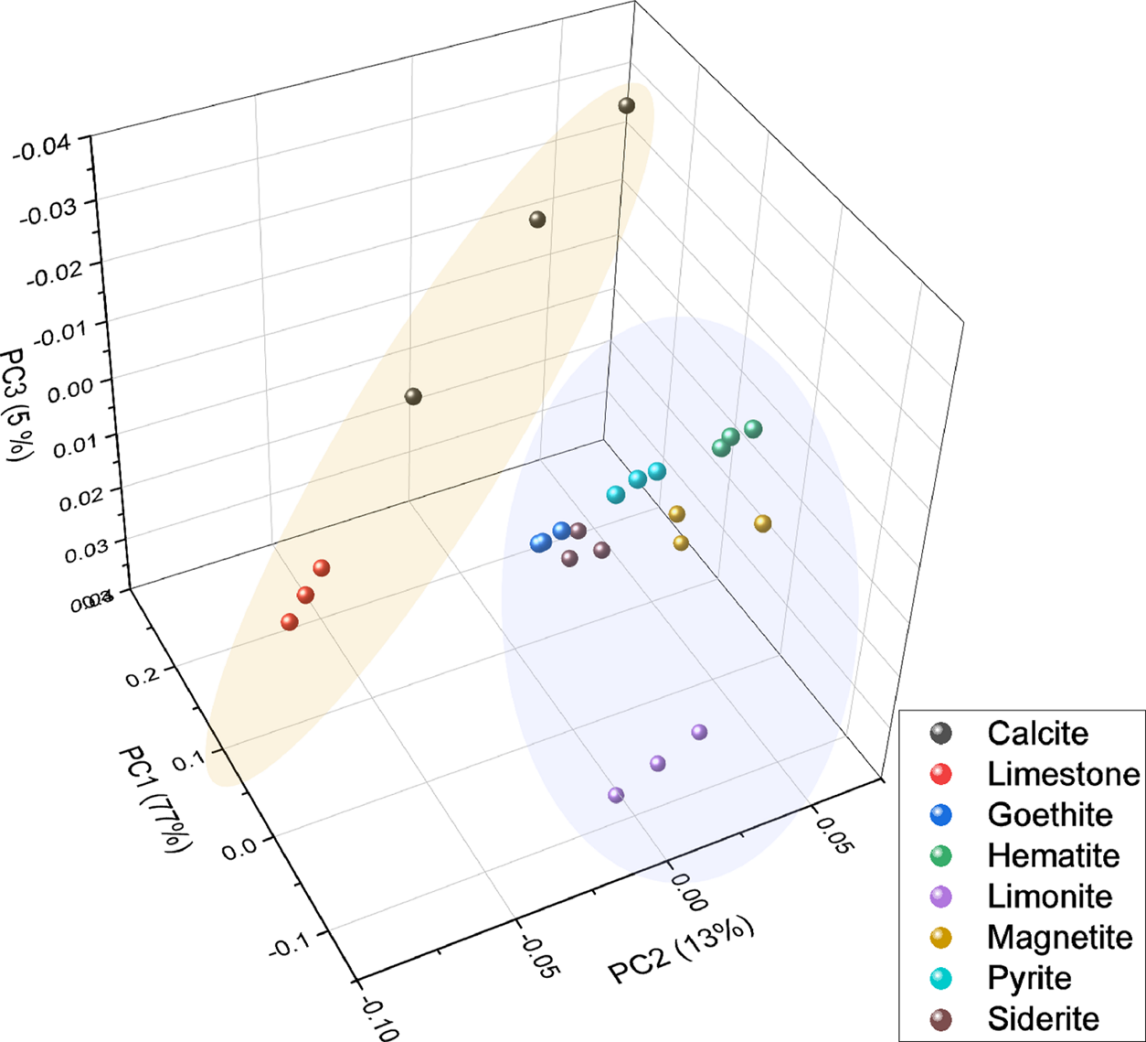
PCA scores for the first three PCs. Cold tones correspond to Fe-rich materials, while warm tones correspond to the Ca-rich subgroup.

Apart from the specified changes, other experimental parameters remained as described in Section 3. In this scenario, lower signals were detected for both LIBS spectra and the acoustic wave. Figure S2 in Supporting Information compares the LIBS emission spectra on Mars-like and Earth conditions for calcite and pyrite. Overall, less intense emission intensity was observed, as well as the signal-to-noise ratio (SNR) strongly decreased in the Mars atmosphere due to changes in the plasma dynamics, decrease in the electronic temperature, and thinner optical plasma density. Table 2 presents the SNR values for two selected emission lines, Ca II at 393 nm and Fe I at 388 nm, in calcite and pyrite, respectively. Moreover, it should be noted that the molecular emission bands were absent in calcite. The most prominent molecular systems featured in Earth LIBS spectra for calcite were CaO/CaOH at 550 nm. Apart from the mentioned physical changes, the pressure and the composition changed; thus, the drop may indicate recombination with dissociated atmospheric O_2_ as the main source of the CaO band. This statement is further supported by the fact that the CO_2_ found in the Martian atmosphere has a higher dissociation energy than O_2_ (532 vs 498 kJ mol^–1^ at 298 K), relinquishing a lower number of O atoms into the plasma. Following the procedure described above, a PCA was carried out on the LIBS data. The spectral matrix was also mean-centered, and the explained variance was obtained, yielding 95% for the first three PCs. Similarly, the Fe-rich and Ca-rich samples were grouped in the 3D scores plot (Figure 8).

**Table 2:** SNR for LIBS and Acoustic Signal in Calcite and Pyrite Samples^a^

	calcite		pyrite	
	Earth	Mars	Earth	Mars
LIBS	525 ± 50	125 ± 40	120 ± 3	11.5 ± 0.5
acoustic	3300 ± 800	35 ± 18	6000 ± 1600	80 ± 20

a LIBS SNR values correspond to the Ca II line at 393 nm (calcite) and Fe I line at 388 nm (pyrite). Acoustic SNR values computed from the peak-to-peak amplitude.

**Figure 9:**
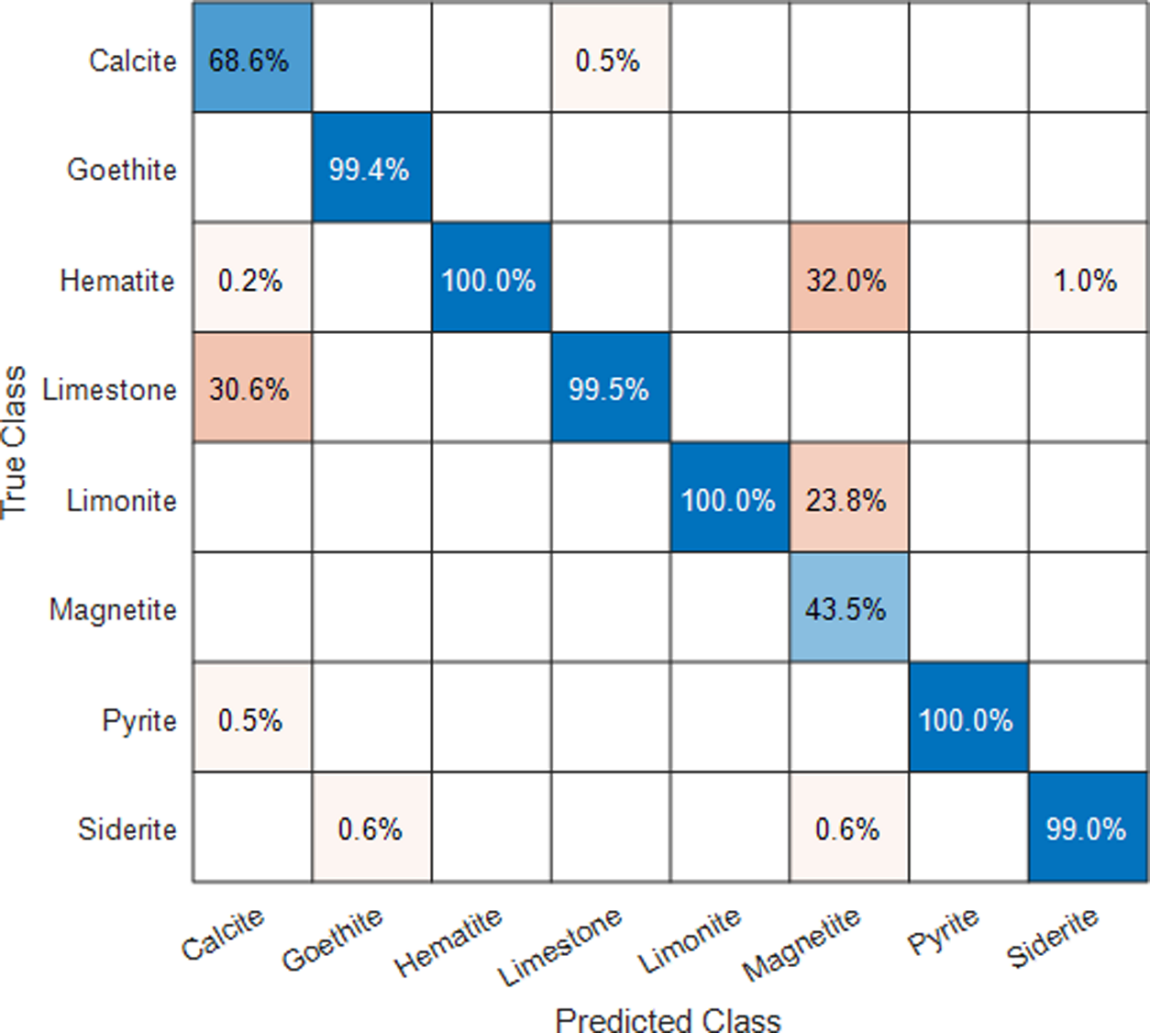
Percentage confusion matrix for the studied samples measured in Mars-like atmosphere.

Acoustic waves were also measured and processed as previously, exhibiting a considerably reduced signal intensity. Due to the pronounced decrease of the acoustic signal (almost 2 orders of magnitude) and the unavoidable decrease of the SNR (Table 2) in the simulated Martian atmosphere, most of the Ca samples, which had the lowest emission intensity in the previous study, did not provide a statistically detectable signal. Although this reduced the sample set, the mid-level data fusion strategy allowed no cross-predictions in the class assignation between groups (Figure 9), so the study of the Fe-rich subgroup was expected to provide an idea of the behavior of the data fusion under different conditions. Limestone and calcite data were preserved, as their signal was larger than the microphone threshold. To improve the identification of the peaks by the algorithm, a bandpass filter was applied between 500 and 20 000 Hz, which eliminated part of the detected noise and allowed the identification of the signal coming from the plasma. Following the workflow presented in Figure 2, LIBS-PCA scores values and the acoustic features were concatenated and an LDA was applied in parallel to the procedure followed in the previous section. The success rate for the data fusion treatment was lower in Mars-like atmosphere than under Earth conditions (Table 3); however, the results obtained still showed a promising perspective for the fusion treatment. Lower SNR values were identified as one of the main causes of the lower hit rate given by the model besides the fading of some spectral features (such as the molecular emission) explained above. The confusion matrix for Martian data is presented in Figure 9. Following results in the terrestrial atmosphere, the cross-predictions between limestone and calcite were expected due to the composition of the samples. This same trend can also be found for the limonite–magnetite pair, as previously seen. Cross-prediction was also found for hematite-magnetite. In the work by Chide et al.,^36^ laser-induced phase transition from hematite to magnetite was observed; these thin layers along SNR collapse could lead to the generation of false predictions.

**Table 3:** LDA Hit Rate Obtained in Mars-like Atmosphere of the New Descriptor (Fusion), LIBS, and Acoustic Data

	LIBS (%)	LIBS-PCA (%)	acoustic (%)	fusion (%)
training	77	100	97	100
validation	73	85	81	89

## Conclusions

Herein, a data fusion strategy merging simultaneously acquired LIBS and laser-induced acoustic data was developed to improve the discrimination of spectrally similar minerals in a remote LIBS configuration. A new descriptor used in a mid-level fusion approach was able to improve the discrimination success rate, via LDA, compared to LIBS alone on the two mineral families studied, i.e., Fe-based and Ca-based rocks. The combination of LIBS-PCA and acoustics provided an easily explainable descriptor with a few variables, which avoided the handling of complex data arrays. This combination was based on the fusion of scores values obtained from LIBS data, and the identified acoustic features in time domain, viz., peak-to-peak amplitude, and intraposition amplitude slope. Once validated under Earth conditions, the approach was tested for robustness under Martian conditions. Upon comparison to only LIBS or only acoustic datasets, the new descriptor also improved the discrimination hit rate in the Mars-like atmosphere. The success classification is increased from 90% (LIBS-PCA) and from 77% (acoustic) to 92% using the fused descriptor under Earth atmospheric conditions, while in Mars-like atmosphere, the increment goes from 85/81% (LIBS-PCA/acoustics) to 89% (mid-level fusion).

While this paper constitutes a preliminary attempt at fusing LIBS and acoustics to maximize the information yielded by analysis events, its possible implementation in the analysis of samples in an open environment must be carried out carefully, especially when acoustic data are involved. The presence of echoes or interferences can modify the time domain signal if the path difference is small enough to interfere (either constructively or destructively) in the prompt acoustic wave, thus changing the peak-to-peak values. One remaining issue is the slight decrease of the hit rate in Martian atmosphere data. Instruments with a better signal-to-noise ratio (more sensitive microphones or the use of an intensified CCD, for instance) could improve these results and bring them on a par with those obtained in the terrestrial atmosphere.

## Supporting Information Available

Confusion matrices for LIBS-PCA and acoustic inputs (Figure S1) and comparative mineral LIBS spectra in Earth and Mars-like atmosphere (Figure S2) (PDF)

## References

[ref1] De Giacomo A.; Hermann J. Laser-Induced Plasma Emission: From Atomic to Molecular Spectra. J. Phys. D: Appl. Phys. 2017, 50, 183002 10.1088/1361-6463/aa6585.

[ref2] Bogaerts A.; Chen Z.; Gijbels R.; et al. Laser Ablation for Analytical Sampling: What Can We Learn from Modeling?. Spectrochim. Acta, Part B 2003, 58, 1867–1893. 10.1016/j.sab.2003.08.004.

[ref3] Hahn D. W.; Omenetto N. Laser-Induced Breakdown Spectroscopy (LIBS), Part I: Review of Basic Diagnostics and Plasmaparticle Interactions: Still-Challenging Issues within the Analytical Plasma Community. Appl. Spectrosc. 2010, 64, 335–366. 10.1366/000370210793561691. 21144145

[ref4] Hahn D. W.; Omenetto N. Laser-Induced Breakdown Spectroscopy (LIBS), Part II: Review of Instrumental and Methodological Approaches to Material Analysis and Applications to Different Fields. Appl. Spectrosc. 2012, 66, 347–419. 10.1366/11-06574. 22449322

[ref5] Laserna J.; Vadillo J. M.; Purohit P. Laser-Induced Breakdown Spectroscopy (LIBS): Fast, Effective, and Agile Leading Edge Analytical Technology. Appl. Spectrosc. 2018, 72, 35–50. 10.1177/0003702818791926. 30265142

[ref6] Purohit P.; Fortes F. J.; Laserna J. J. Spectral Identification in the Attogram Regime through Laser-Induced Emission of Single Optically Trapped Nanoparticles in Air. Angew. Chem., Int. Ed. 2017, 56, 14178–14182. 10.1002/anie.201708870. 28877398

[ref7] Fortes F. J.; Moros J.; Lucena P.; et al. Laser-Induced Breakdown Spectroscopy. Anal. Chem. 2013, 85, 640–669. 10.1021/ac303220r. 23137185

[ref8] Alvarez-Llamas C.; Roux C.; Musset O. A Compact, High-Efficiency, Quasi-Continuous Wave Mini-Stack Diode Pumped, Actively Q-Switched Laser Source for Laser-Induced Breakdown Spectroscopy. Spectrochim. Acta, Part B 2018, 148, 118–128. 10.1016/j.sab.2018.06.012.

[ref9] Guirado S.; Fortes F. J.; Laserna J. J. Elemental Analysis of Materials in an Underwater Archeological Shipwreck Using a Novel Remote Laser-Induced Breakdown Spectroscopy System. Talanta 2015, 137, 182–188. 10.1016/j.talanta.2015.01.033. 25770623

[ref10] Roux C. P. M.; Rakovský J.; Musset O.; et al. In Situ Laser Induced Breakdown Spectroscopy as a Tool to Discriminate Volcanic Rocks and Magmatic Series, Iceland. Spectrochim. Acta, Part B 2015, 103–104, 63–69. 10.1016/j.sab.2014.11.013.

[ref11] Maurice S.; Clegg S. M.; Wiens R. C.; et al. ChemCam Activities and Discoveries during the Nominal Mission of the Mars Science Laboratory in Gale Crater, Mars. J. Anal. At. Spectrom. 2016, 31, 863–889. 10.1039/c5ja00417a.

[ref12] Wiens R. C.; Maurice S.; Robinson S. H.; et al. The SuperCam Instrument Suite on the NASA Mars 2020 Rover: Body Unit and Combined System Tests. Space Sci. Rev. 2021, 217, 4 10.1007/s11214-020-00777-5. 33380752 PMC7752893

[ref13] Maurice S.; Wiens R. C.; Bernardi P.; et al. The SuperCam Instrument Suite on the Mars 2020 Rover: Science Objectives and Mast-Unit Description. Space Sci. Rev. 2021, 217, 47 10.1007/s11214-021-00807-w. PMC7752893 33380752

[ref14] Chide B.; Maurice S.; Murdoch N.; et al. Listening to Laser Sparks: A Link between Laser-Induced Breakdown Spectroscopy, Acoustic Measurements and Crater Morphology. Spectrochim. Acta, Part B 2019, 153, 50–60. 10.1016/j.sab.2019.01.008.

[ref15] Conesa S.; Palanco S.; Laserna J. J. Acoustic and Optical Emission during Laser-Induced Plasma Formation. Spectrochim. Acta, Part B 2004, 59, 1395–1401. 10.1016/j.sab.2004.06.004.

[ref16] Chaléard C.; Mauchien P.; Andre N.; et al. Correction of Matrix Effects in Quantitative Elemental Analysis With Laser Ablation Optical Emission Spectrometry. J. Anal. At. Spectrom. 1997, 12, 183–188. 10.1039/A604456E.

[ref17] Murdoch N.; Chide B.; Lasne J.; et al. Laser-Induced Breakdown Spectroscopy Acoustic Testing of the Mars 2020 Microphone. Planet. Space Sci. 2019, 165, 260–271. 10.1016/j.pss.2018.09.009.

[ref18] Chide B.; Maurice S.; Cousin A.; et al. Recording Laser-Induced Sparks on Mars with the SuperCam Microphone. Spectrochim. Acta, Part B 2020, 174, 106000 10.1016/j.sab.2020.106000.

[ref19] Alvarez-Llamas C.; Purohit P.; Moros J.In A Multipurpose Thermal Vacuum Chamber for Planetary Research Compatible with Stand-Off Laser Spectroscopies, 52nd Lunar and Planetary Science Conference No. 2548, 2021; p 2330. https://www.hou.usra.edu/meetings/lpsc2021/pdf/2330.pdf.

[ref20] Klingelhöfer G.; DeGrave E.; Morris R. V.; et al. Mössbauer Spectroscopy on Mars: Goethite in the Columbia Hills at Gusev Crater. Hyperfine Interact. 2005, 166, 549–554. 10.1007/s10751-006-9329-y.

[ref21] Niles P. B.; Catling D. C.; Berger G.; et al. Geochemistry of Carbonates on Mars: Implications for Climate History and Nature of Aqueous Environments. Space Sci. Rev. 2013, 174, 301–328. 10.1007/s11214-012-9940-y.

[ref22] McLennan S. M.; Anderson R. B.; Bell J. F.; et al. Elemental Geochemistry of Sedimentary Rocks at Yellowknife Bay, Gale Crater, Mars. Science 2014, 343, 1244734 10.1126/science.1244734. 24324274

[ref23] Kah L. C.; Stack K. M.; Eigenbrode J. L.; et al. Syndepositional Precipitation of Calcium Sulfate in Gale Crater, Mars. Terra Nova 2018, 30, 431–439. 10.1111/ter.12359.

[ref24] Vaniman D. T.; Bish D. L.; Ming D. W.; et al. Mineralogy of a Mudstone at Yellowknife Bay, Gale Crater, Mars. Science 2014, 343, 1243480 10.1126/science.1243480. 24324271

[ref25] Mittlefehldt D. W. ALH84001, a Cumulate Orthopyroxenite Member of the Martian Meteorite Clan. Meteoritics 1994, 29, 214–221. 10.1111/j.1945-5100.1994.tb00673.x.

[ref26] Boynton W. V.; Ming D. W.; Kounaves S. P.; et al. Evidence for Calcium Carbonate at the Mars Phoenix Landing Site. Science 2009, 325, 61–64. 10.1126/science.1172768. 19574384

[ref27] Borràs E.; Ferré J.; Boqué R.; et al. Data Fusion Methodologies for Food and Beverage Authentication and Quality Assessment—A Review. Anal. Chim. Acta 2015, 891, 1–14. 10.1016/j.aca.2015.04.042. 26388360

[ref28] Brereton R. G. Applied Chemometrics for Scientists; John Wiley & Sons, Ltd, 2007.

[ref29] Bro R.; Smilde A. K. Principal Component Analysis. Anal. Methods 2014, 6, 2812–2831. 10.1039/c3ay41907j.

[ref30] Brereton R. G. Chemometrics for Pattern Recognition; John Wiley & Sons, Ltd, 2009.

[ref31] Harri A. M.; Genzer M.; Kemppinen O.; et al. Pressure Observations by the Curiosity Rover: Initial Results. J. Geophys. Res.: Planets 2014, 119, 82–92. 10.1002/2013JE004423. PMC4508910 26213667

[ref32] Hunt G. E. Planetary Atmospheres. Nature 1979, 279, 354 10.1038/279354a0.

[ref33] Colao F.; Fantoni R.; Lazic V.; et al. LIBS Application for Analyses of Martian Crust Analogues: Search for the Optimal Experimental Parameters in Air and CO2 Atmosphere. Appl. Phys. A: Mater. Sci. Process. 2004, 79, 143–152. 10.1007/s00339-003-2262-x.

[ref34] Schröder S.; Rammelkamp K.; Vogt D. S.; et al. Contribution of a Martian Atmosphere to Laser-Induced Breakdown Spectroscopy (LIBS) Data and Testing Its Emission Characteristics for Normalization Applications. Icarus 2019, 325, 1–15. 10.1016/j.icarus.2019.02.017.

[ref35] Brennetot R.; Lacour J. L.; Vors E.; et al. Mars Analysis by Laser-Induced Breakdown Spectroscopy (MALIS): Influence of Mars Atmosphere on Plasma Emission and Study of Factors Influencing Plasma Emission with the Use of Doehlert Designs. Appl. Spectrosc. 2003, 57, 744–752. 10.1366/000370203322102816. 14658651

[ref36] Chide B.; Beyssac O.; Benzerara K. Acoustic Monitoring of Laser-Induced Phase Transition in Minerals, 51st Lunar and Planetary Science Conference,2020; p 1818.

