# Supplementary material for: LIBS-Acoustic Mid-Level Fusion Scheme for Mineral Differentiation under Terrestrial and Martian Atmospheric Conditions
Source: Anal Chem. 2022 Jan 12;94(3):1840–9. doi: 10.1021/acs.analchem.1c04792 (PMC8893358; doi:10.1021/acs.analchem.1c04792)

LIBS-Acoustic Mid-Level Fusion Scheme for Mineral Differentiation under Terrestrial and Martian Atmospheric Conditions

César Alvarez-Llamas‡, Pablo Purohit‡, Javier Moros and Javier Laserna*

UMALASERLAB, Departamento de Química Analítica, Universidad de Málaga, C/ Jiménez Fraud 4, Málaga, 29010, Spain

Table of Contents

Additional Figures……………………… S2

Figure S1……………………….. S2

Figure S2……………………….. S3

Figure S1: a) Confusion matrix from the PCA-LIBS data. b) Confusion matrix from Acoustic feature

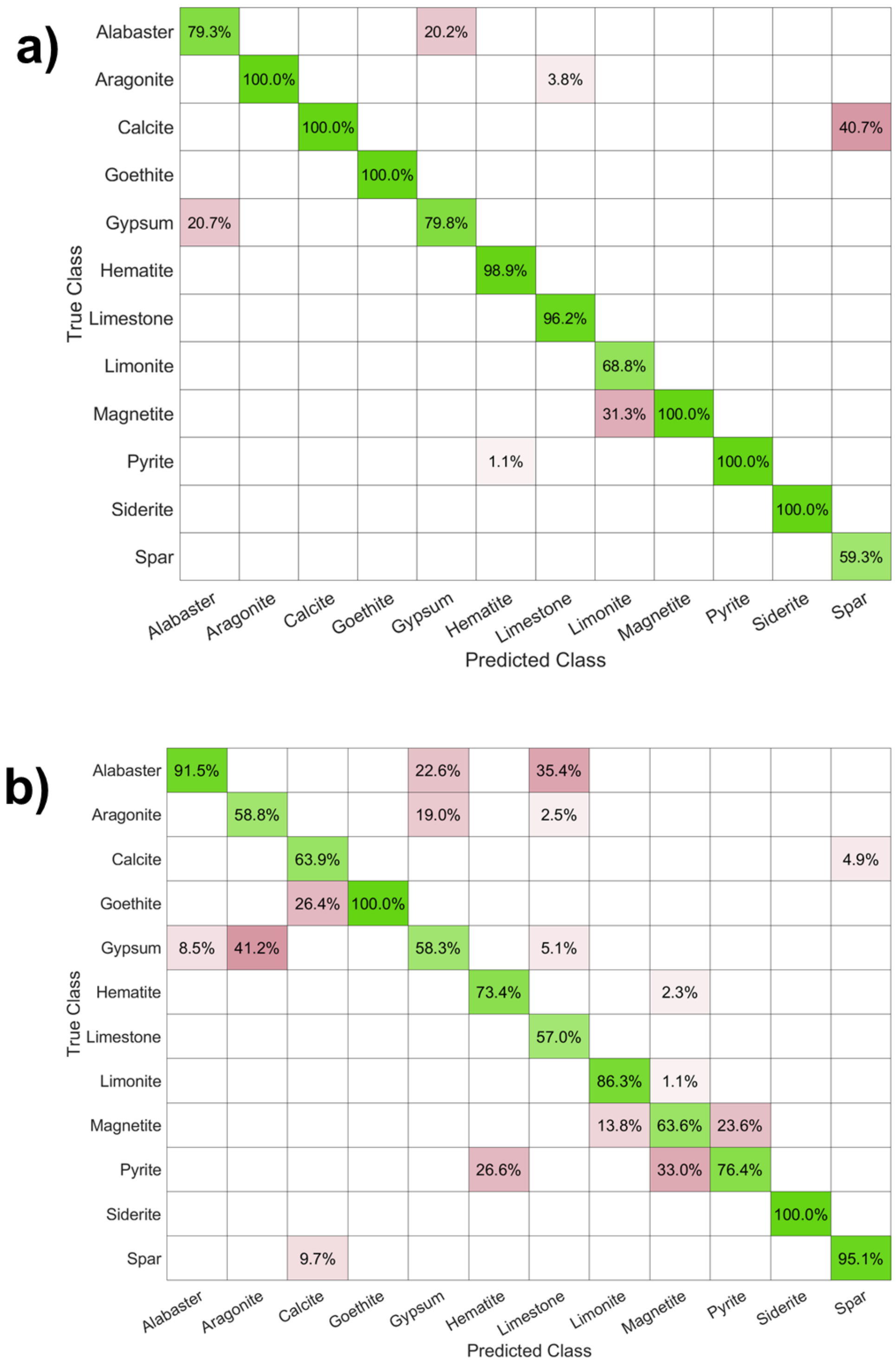

Figure S2. LIBS spectra for Pyrite and Calcite in Earth and Mars-like atmospheres

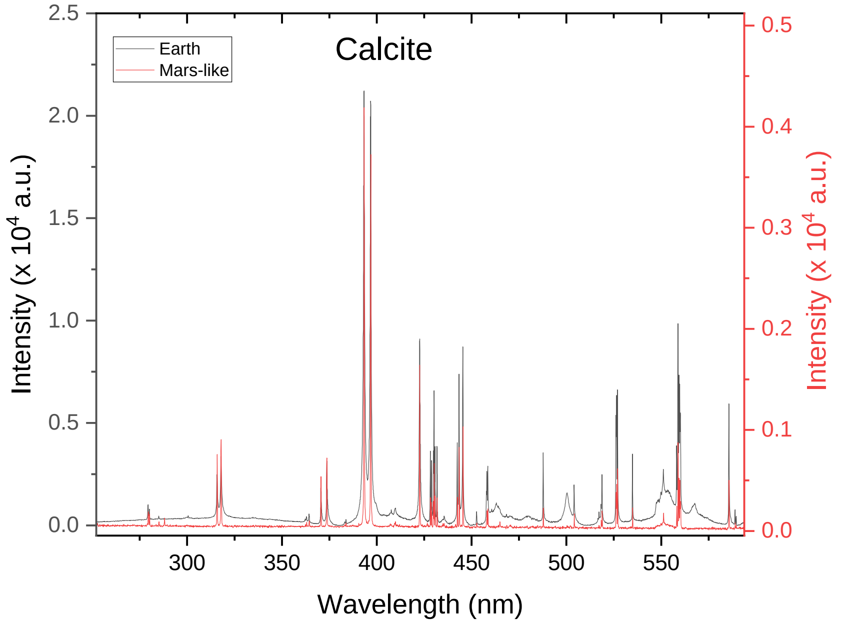

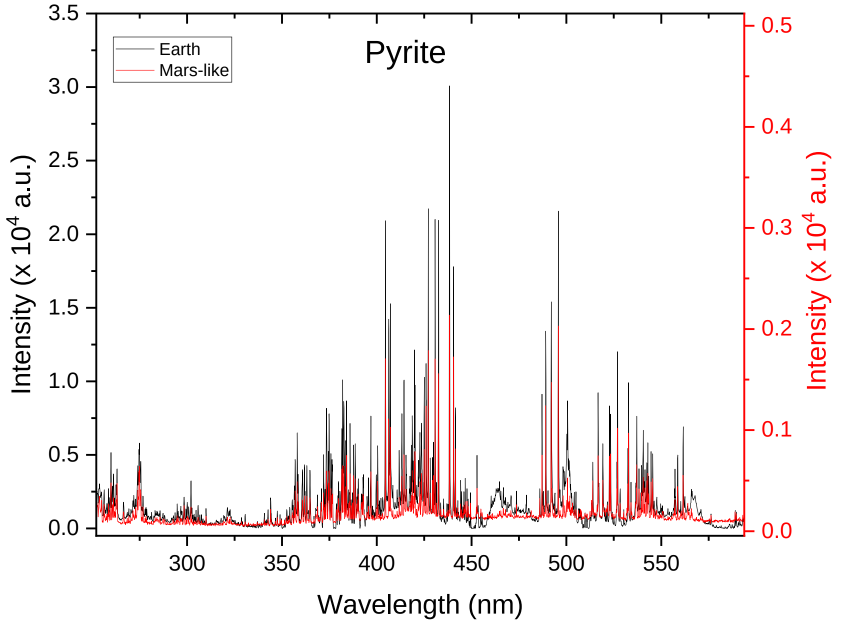

Supplement: Supplementary file 1 — ac1c04792_si_001.pdf [file ac1c04792_si_001.pdf]
